# Moderators of the Effectiveness of UPcomplish on Office Workers’ Sedentary Behaviour, Quality of Life, and Psychosocial Determinants: A Stepped Wedge Design

**DOI:** 10.1007/s12529-022-10147-w

**Published:** 2023-01-31

**Authors:** Nathalie M. Berninger, Rik Crutzen, Robert A. C. Ruiter, Gerjo Kok, Guy Plasqui, Gill A. ten Hoor

**Affiliations:** 1 Department of Work and Social Psychology, Faculty of Psychology and Neuroscience, Maastricht University, Universiteitssingel 40, PO Box 616, 6200 MD Maastricht, The Netherlands; 2 Department of Health Promotion, CAPHRI, Maastricht Universit, Maastricht, the Netherlands; 3 Department of Human Biology and Movement Sciences, Nutrition and Translational Research in Metabolism, Maastricht University, Maastricht, the Netherlands

**Keywords:** Sedentary behaviour, Intervention Mapping, Quality of life, Vitality, Office workers

## Abstract

**Background:**

In the earlier developed and evaluated 12-week UPcomplish intervention, the aim was to reduce sedentary behaviour (SB) among office workers and increase their quality of life (QoL). In the current study, we explored moderators of effectiveness.

**Method:**

We applied a stepped wedge design with five intervention groups starting with time lags of seven weeks (*n* = 142, 96 females). Participants wore the VitaBit to continuously measure SB and received surveys about QoL and psychosocial determinants at the beginning, middle, and end of the intervention. We regressed baseline participant characteristics and behaviours onto intra-individual improvements (centred around calendar week means) in determinants, SB, performance objectives, and QoL.

**Results:**

Those scoring high in baseline intention, task performance, stress, vitality, and emotional well-being improved less in these variables. Baseline stress (*β* =  − 0.05 [SE = 0.01; 95% CI =  − 0.08, − 0.02; *p*_corrected_ = .02]) and emotional well-being (*β* = 0.02 [SE = 0.01; 95% CI = 0.01, 0.03; *p*_corrected_ = .02]) were associated with improvement in contextual performance. Baseline attitude (*β* =  − 12.92 [SE = 3.93; 95% CI =  − 20.80, − 5.04; *p*_corrected_ = .02]) and perceived behavioural control (PBC; *β* =  − 9.27 [SE = 3.04; 95% CI =  − 15.37, − 3.16; *p*_corrected_ = .03]) were negatively associated with improvements in emotional well-being. Post hoc analyses with a sub-group scoring lower in determinants revealed that improvement in PBC was positively associated with SB registration.

**Conclusion:**

Participants scoring low in baseline determinants might profit from UPcomplish via an increase in PBC. In combination with changes within organizations (e.g. the implementation of standing desks), UPcomplish might potentially reduce SB.

**Trial Registration:**

NL7503 — registered 1 February 2019.

## Highlights

- Among less motivated participants, improving self-efficacy predicts improvement in sitting.
- Sedentary behavior might be more automatic and habitual and less intentional.
- Sitting less probably needs to be facilitated with environmental and cultural changes.

## Introduction

In recent decades, there has been an exponential growth of office work in Western societies, dominated by sedentary activities [1]. Sedentary behaviour (SB) includes sitting, lying, or reclining activities with low energy expenditure [2]. Employees in high-income countries across the globe were found to sit for about 60% of their days [1]. In a study in Norway, Chau and colleagues found that compared to employees exhibiting more active jobs involving more walking and lifting, office workers have 35% increased mortality rates [3]. One of the reasons for this is that independently of leisure time exercise, SB increases the risk for cardio-metabolic diseases [4, 5]. Despite increasing numbers of interventions to reduce office workers’ SB, there is mixed evidence of their effectiveness [6].

Although interventions that involved environmental restructuring, i.e. the implementation of standing desks, or that involved personal coaching, have been found to be effective in reducing SB, they are cost-intensive [7–10]. For a large-scale implementation, low-cost interventions are needed. However, current interventions that are low-cost and focus on changing attitudes as determinants of behaviour without environmental or workplace policy changes supporting behaviour change show mixed effects [11, 12]. Therefore, we developed a low-cost alternative to personal coaching, UPcomplish, involving personal coaches supporting participants with automated content [13]. During the coaching, participants wear the VitaBit sensor [14]. The VitaBit toolkit includes the sensor measuring physical behaviour (i.e. SB and physical activity), a mobile phone monitoring application, and a computer portal, where participants can set goals and compete with others. VitaBit also provides a coaching portal, where coaches, if they are authorized, can retrieve participants’ physical behaviour data.

**Fig. 1:**
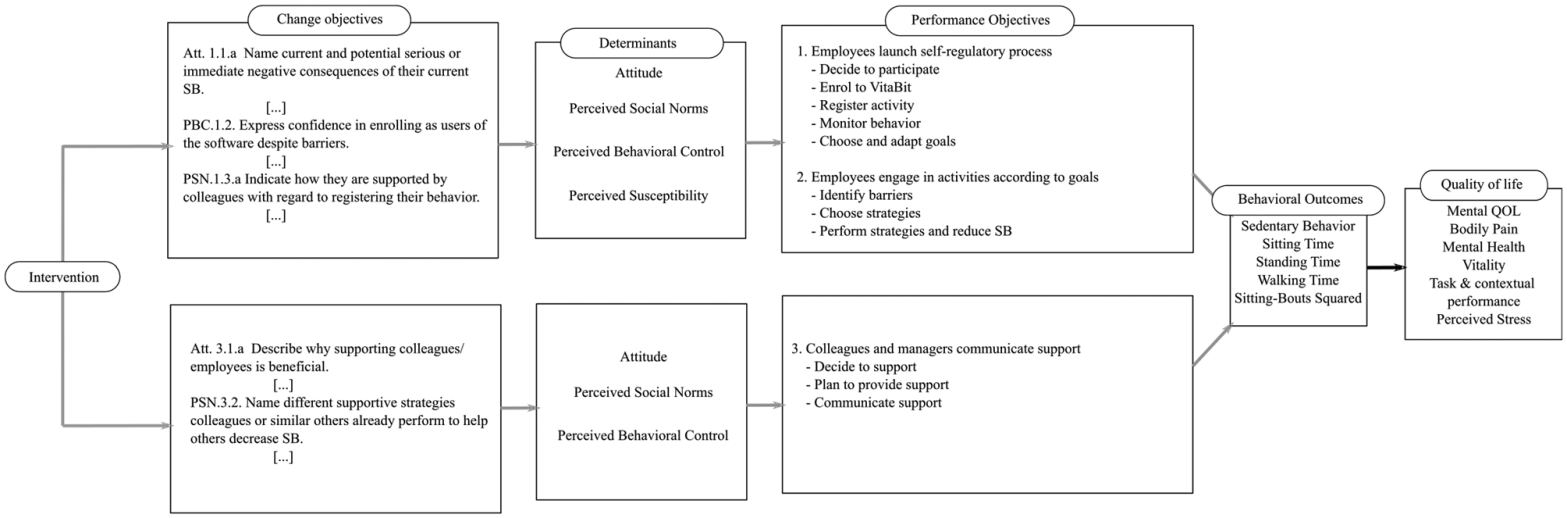
Illustration of the logic model of the UPcomplish and VitaBit intervention. The performance objectives on the individual level are shown in the upper branch, the ones on the interpersonal level are shown in the lower branch

By employing Intervention Mapping (IM), a framework for the systematic development of behaviour change interventions [15], we used findings from behaviour change theories, the empirical literature, and our research data to systematically develop UPcomplish. The result was a logic model of the intervention, of which an excerpt is shown in Fig. 1. It depicts the causal mechanisms from the practical applications of the UPcomplish intervention to the behavioural outcome, i.e. reducing SB. For example, tailored feedback on the achievement of goals, combined with positive reinforcement, is theorized to change the psychosocial determinant attitude. One of the underlying attitudinal beliefs (~ Change objectives) being targeted by this feedback is as follows: “Indicate that the number of resources (time, skills) that will need to be invested to perform certain strategies [being suggested to reduce sitting] will be worthwhile as it will lead to positive outcomes”. The logic model assumes that changing this attitudinal belief will help to reduce SB [13].

UPcomplish is a data-driven, tailored, and motivational intervention involving the VitaBit toolkit that allows for the self-monitoring of SB. We implemented UPcomplish among 15 workplace sites to investigate its effectiveness. For the effect evaluation, we had expected the intervention to be effective in reducing daily sitting proportion and prolonged sitting as well as in increasing quality of life (QoL; i.e. vitality, performance, and well-being). Yet, compared to the VitaBit-only baseline phases (i.e. control condition), we did not find significant improvements. Both between and within participants did the UPcomplish intervention reduce SB reduce or increase QoL [16]. Possible reasons for this may be a recruitment bias among the intervention population (e.g. only employees being motivated volunteered), but also unexpected deviations from the logic model of change underlying the intervention (Fig. 1) [13]. For example, post hoc analyses found that improvements in the psychosocial determinants were not associated with improvements in SB, and improvements in SB were not associated with improvements in QoL. It might be that either SB among office workers is less of a reasoned action than we assumed or that certain sub-groups of participants engaged more in the intervention, and profited from improvements in determinants, in SB, or in QoL. The intervention population was dominated by females (68%), and the participants reported high baseline QoL and psychosocial determinants. These and other baseline and participants’ characteristics might have been factors that moderated the effectiveness of UPcomplish.

The purpose of this study is to explore potential moderators of the effectiveness of UPcomplish. Effectiveness refers to improvements in psychosocial determinants, in SB, and in QoL, as well as performance objectives (i.e. average registering, monitoring, and engagement with coach) [15], which serve as dependent variables. As independent variables, firstly, participant characteristics such as gender, age, body mass index (BMI), or employment status are explored. Secondly, we assumed that low baseline SB, high moderate-to-vigorous physical activity (MVPA), positive baseline determinants, and high baseline QoL result in lower potential for improvement and, therefore, less effectiveness of the intervention. Thirdly, the intervention messages might not have been accepted, read, or understood. Instead of using a randomized control trial, the data were gathered using a stepped wedge design with continuous recruitment. As a result, we received annual spread data, increased statistical power, and avoided having a waiting control group (which is often associated with compliance issues) [17].

## Methods

The study was pre-registered under: NL7503 (https://www.trialregister.nl/trial/7503). The protocol of the intervention, with more details about the design, has been published elsewhere [13]. Additional material, the raw data, and the R scripts are fully disclosed in the supplementary material https://osf.io/qzp9m/. This manuscript adheres to the Consolidated Standards of Reporting Trials (CONSORT) checklist of information to include when reporting a stepped wedge cluster randomized trial [18].

### Study Design and Sample

We had five intervention groups each including participants from 2 to 5 different companies (i.e. sub-groups). The groups started with time lags of about 7 weeks, each worksite receiving a kick-off meeting with a minimum of 5 employees per kick-off. This was followed by the baseline, VitaBit-only week, and the 12-week UPcomplish intervention. The eligibility criteria included that participants were able to walk and stand, that they were willing to download the VitaBit smartphone application, that they were office workers, and that they were able to speak and understand German. If any of the inclusion criteria were not met, participants were excluded.

**Fig. 2:**
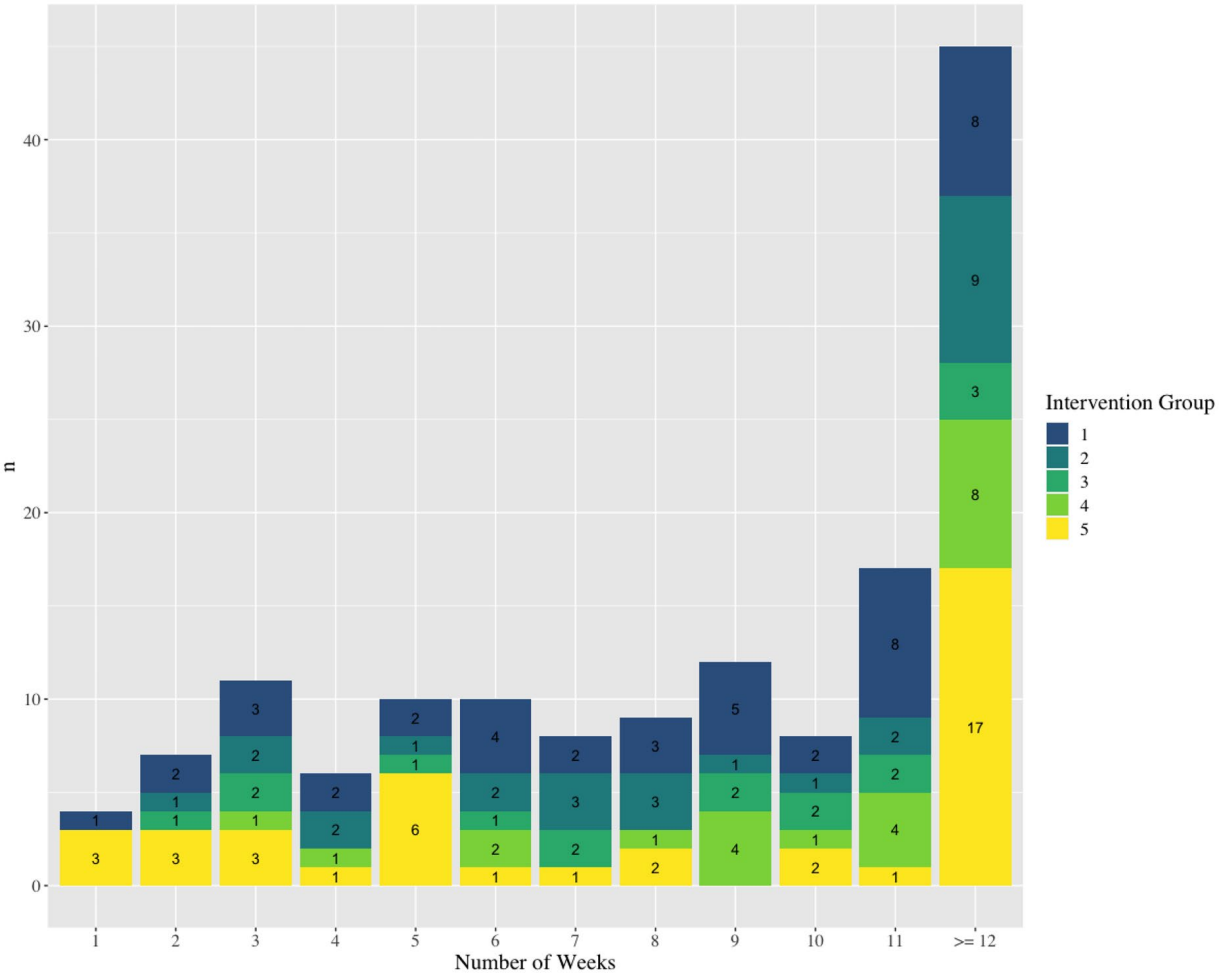
Number of participants per intervention group per number of weeks having collected VitaBit

**Fig. 3:**
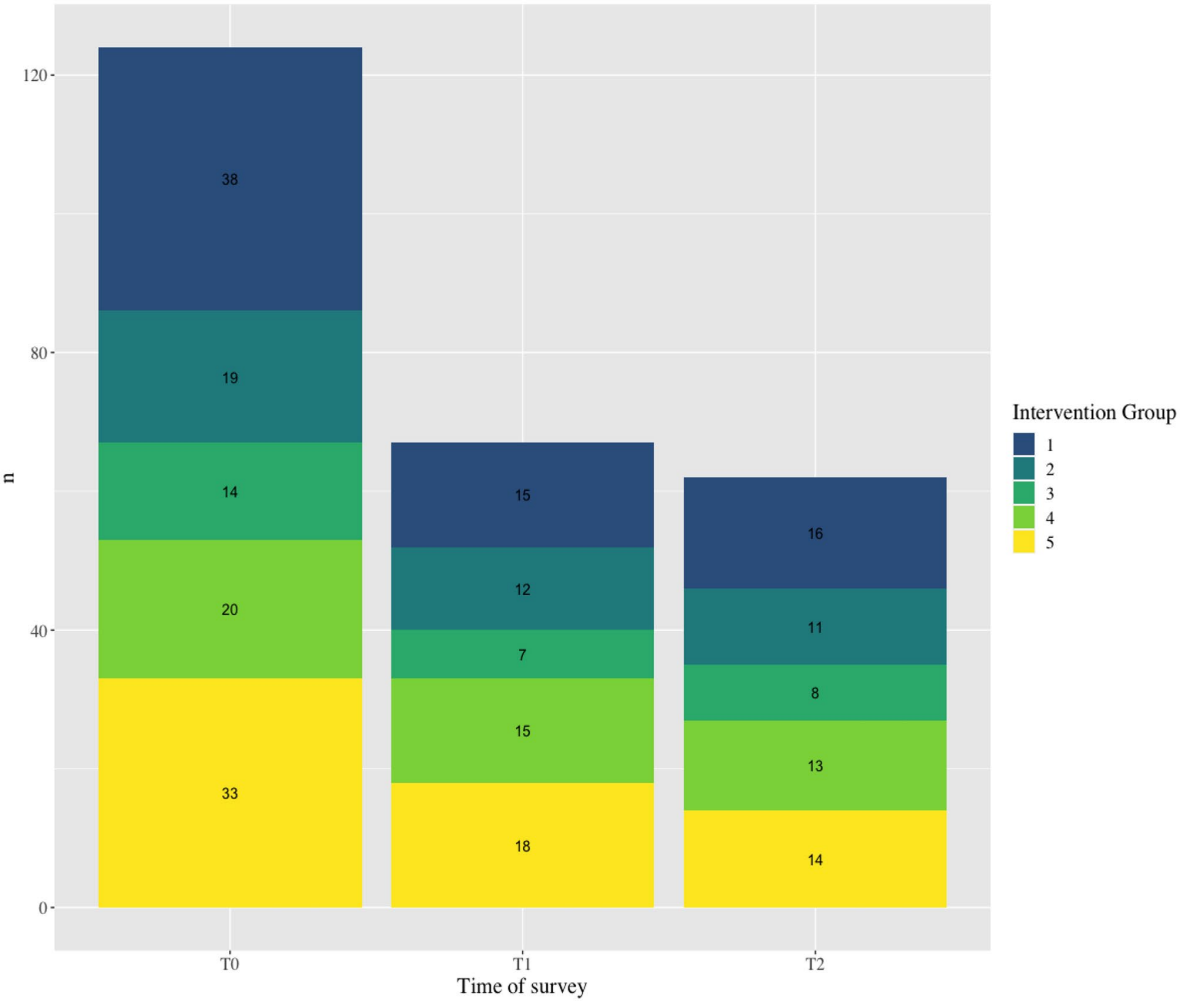
Number of participants that filled out the survey at baseline (T0), in the middle of the intervention (T1), and directly after the intervention (T2)

VitaBit Software provided us with 200 devices, which we could use for the evaluation study (May 2019–January 2020). Assuming five intervention groups of 40 participants each and a drop-out of 20% (32 participants per group, one group serving as both baseline and control), we conducted power calculations with an expected sample size of *N* = 192 and a Cohen’s *d* estimate of 0.5. The population effect size would very likely (95%) be somewhere between 0.21 and 0.79, which we considered being sufficiently accurate [13]. We recruited participants from German companies in multiple industries (e.g. public service, education, and automotive). Of the 193 eligible participants who communicated interest in participating, 150 participants created a VitaBit account, and 142 wore their VitaBit at baseline. The flow of the participants in the intervention is shown in Fig. 2: 45 participants wore the VitaBit device for 12 weeks or longer, whereas 38 participants collected less than 6 weeks of VitaBit data and were therefore excluded from the analyses of the current study. The number of participants that filled out the surveys is illustrated in Fig. 3. The baseline survey (T0) was filled out by 129 (91%), the mid-evaluation survey (T1) by 67 (47%), and the end evaluation survey (T2) by 62 (44%) participants.

Participants could refuse their participation at all times, without giving a reason. This study and the consent procedure were approved by the Ethics Review Committee of the Faculty of Psychology and Neuroscience, Maastricht University, Maastricht, The Netherlands (ERCPN-188_11_02_2018). The trial was pre-registered in the Netherlands Trial Register under: NL7503 (https://www.trialregister.nl/trial/7503).

### Procedure

Flyers with information about the study (incl. inclusion criteria, benefits to expect, and what to do) were distributed among German companies and potential participants (i.e. employees) who, if they were interested, further forwarded the flyers. If the management agreed, the employees could participate. Emails with an invitation to the personal kick-off meeting, instructions on creating a VitaBit account, and the information sheet were sent to volunteering participants. The kick-offs, which took place in the participants’ companies, took between 35 and 60 min and included an introduction round, information about SB, the intervention, and the VitaBit toolkit. Additionally, participants were supported to pair the VitaBit devices with their smartphones. After written informed consent, participants started wearing the device. The first week was the baseline, VitaBit-only week. This was followed by the 12-week intervention. Participants who were interested, but unable to attend the kick-offs, received all information via email. At baseline, in week 6, and directly after the intervention, participants received surveys on QoL and determinants. After the intervention, everyone received an individual and a group (i.e. company) report and a VitaBit voucher as compensation. The devices were collected earliest 4 weeks after the end of the intervention.

### Intervention

The intervention consists of two components: the VitaBit mobile phone application (app) and UPcomplish. The VitaBit app serves as monitoring tool, providing information about current SB, standing, and physical activity, and showing the user’s personal goals. UPcomplish serves as motivational support and includes 14 feedback messages (FBMs) that are sent to participants via their preferred channel (e.g. WhatsApp, email). In the first 6 weeks, the FBMs were sent twice, and, as of week 6, once per week. They were tailored to individuals’ physical behaviour, their goals (set during the kick-off and adapted after the first week if too easy or too difficult), and their perceived barriers. If participants did not drop out, they received (1) a FBM, (2) a reminder if they forgot to wear their device, or (3) no message in case of a holiday. In the latter two cases, the upcoming FBMs were delivered delayed. The last two FBMs were not delayed and were delivered to all participants having data at the concerning point in time. Thereby, all participants could compete with each other and receive tips on how to keep the new habits. The FBMs included support in goal setting, goal adjustment, breaking down the goal to graded sub-goals, and feedback about the goals. Additionally, they included feedback about SB patterns (e.g. “*On Tuesday afternoon, your sitting periods seem to be specifically long*”). After being asked about their hurdles to sit less (e.g. time constraints, kind of work), the participants received tailored advice on how to overcome these hurdles. Every 2 weeks, they received activity challenges, such as not using the toilet on the same floor. In the end, the coach gave tips on how to sustain the new habits.

### Measures

**Fig. 4:**
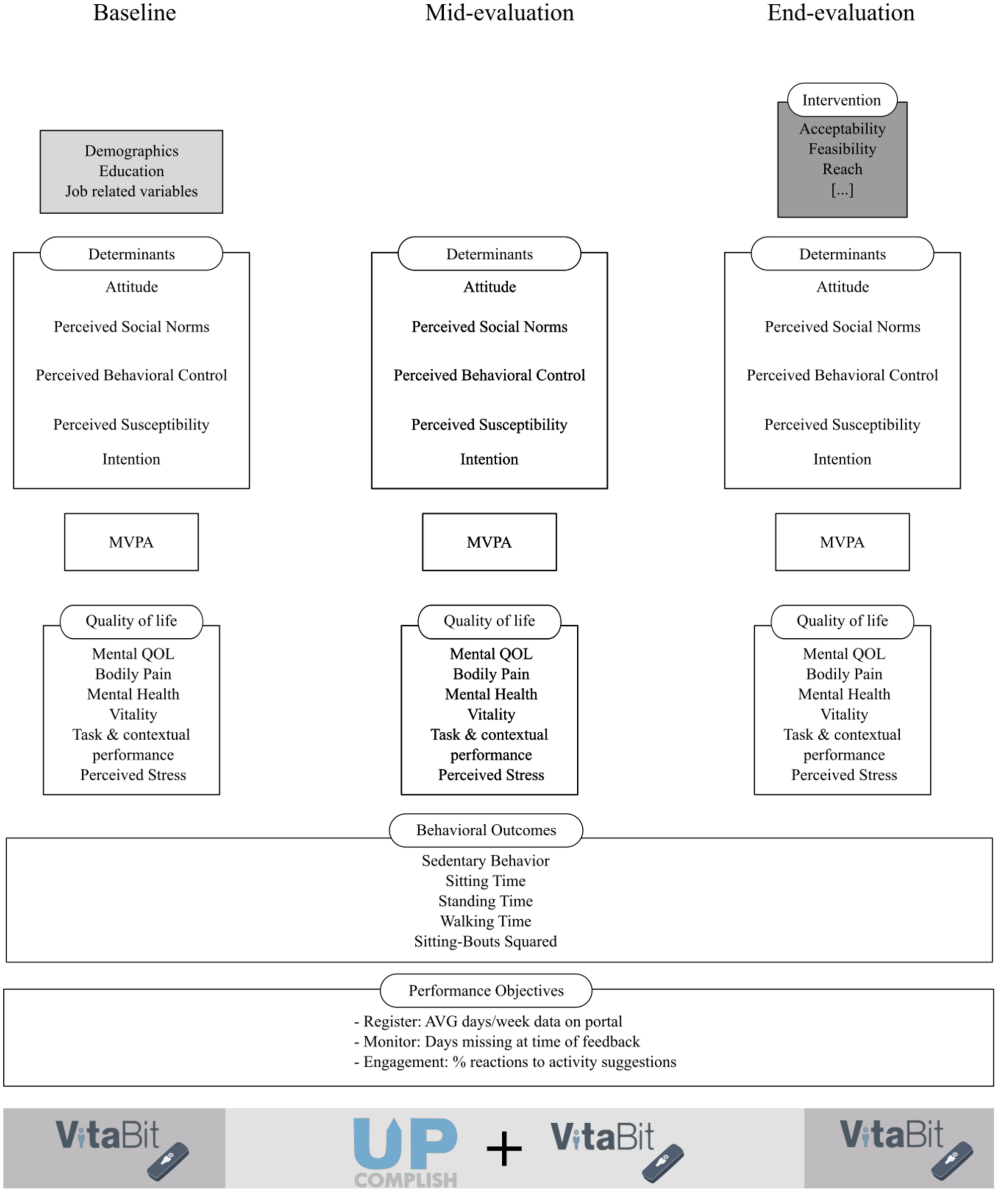
Overview of the measures that were conducted for the evaluation of UPcomplish. At baseline, in week 6, and in the end, surveys on determinants, moderate-to-vigorous physical activity (MVPA), and quality of life were distributed. Physical behaviours (i.e. behavioural outcomes) were continuously measured with the VitaBit. VitaBit data also provided information on performance objectives; for example, how often participants wore the device (i.e. registered behaviour) and opened the app to synchronize their data (i.e. monitored behaviour)

Figure 4 shows the measurements that were implemented during data collection.

#### Continuous Measurements

The VitaBit device (3.9 × 1.4 × 0.9 cm, 4.8 g) measured SB, standing, and activity. It was magnetically attached to clothing fabric at the thigh or placed in trouser pockets. The device samples data with a rate of 33 Hz and an output rate of 30 s, which are stored on the device for at least 30 days. The data on the device are synchronized via Bluetooth with the VitaBit app, before being delivered via mobile Internet to the backend server. The data are stored in a time series database in a pseudonymized way, where they can be downloaded by authorized persons. In a validation study, the device showed a sensitivity of 85.7% and a specificity of 91.2% for sitting [14]. The raw data are in a long format csv file (i.e. each row representing 30 s of a person) and include a user identifier, a time stamp, and three columns for each physical behaviour.

The VitaBit device stores the data even if it is not immediately synchronized with the app. Therefore, information regarding behavioural registering and monitoring could be retrieved indirectly (i.e. performance objectives in Fig. 1). First, the total number of days per week with available data on the VitaBit device provided information on the registration of participants’ SB, i.e. how often the device was worn. Second, to monitor their SB, participants needed to open the VitaBit app and synchronize their data. Since VitaBit did not synchronize the data without manually opening the app, behavioural monitoring could be assessed at the weekly FBMs: The number of days with data that were missing at the time of the FBM was used as a proxy because it gave an estimate on how often the app was opened. More days with data missing would indicate lower monitoring rates.

As a proxy for the third, performance objective (engagement in the coaching, such as action planning and the discovery of barriers), we used the proportion of responses to coaching messages. Participants with more responses to the coaching messages would have higher engagement values than participants who only responded rarely to coaching messages.

#### Online Surveys

Online surveys at baseline (T0), in week 6 (T1), and directly after the intervention (T2) included questions on psychosocial determinants and QoL. The survey at T0 additionally included sociodemographic and job-related variables, and the survey at T2 additionally asked about intervention characteristics. The English version of the Individual Work Performance Questionnaire was translated into German using back-translation [19]. We calculated omegas (*ω*; > 2 items) and Pearson’s correlations (*r*; 2 items) to provide estimates for internal consistency [20, 21]. For all numerical variables, higher survey scores indicated higher values regarding the variable being measured (e.g. higher self-efficacy).

Gender, age, educational level, height, weight, and job-related variables were obtained when the participants created their VitaBit accounts. They could choose between 8 different educational degrees (e.g. high school degree), between 29 different job titles (e.g. sales manager, administrative), and between 17 main company industries (e.g. educational, service). Additionally, in the online survey, they were asked about the usual number of workdays per week (1 item) and their employment status (full-time/part-time; 1 item), and they received questions about sedentary job tasks (5 items). These could be phone calls, computer work, desk work, having meetings, and travelling/visiting clients, such as “*How much - on average per day (in %) - do you estimate you spend on* […] *Phone calls?*” [22].

The questions on acceptability (rated on a 5-point Likert scale; 1 = *I don’t agree*, 5 = *I agree*) encompassed program-related variables (e.g. understandability; 12 items; e.g. “*How much do you agree with the following statements: […] The questions within the recommendations were clear*”), questions about the coach’s advice (e.g. credibility; 7 items), and questions about behavioural maintenance (2 items) [22].

Participants were asked to indicate how much they agreed with statements on SB, which they indicated on a 5-point Likert scale. These statements covered attitude (6 items; e.g. *“*[…] *walking around at work is healthy*”; *ω* = .62), perceived social support (2 items; e.g. *“*[…] *walking around at work is encouraged by my colleagues*”; *r* = .62), perceived behavioural control (PBC; 4 items; e.g. “*I am sure that I can* […] *walk around at work, even though I feel bad, tired, tense or depressed*”; *ω* = .70), and intention (2 items; e.g. “*Are you planning to interrupt long sitting periods at work with* […] *walking breaks?*”; *r* = .43) [22]. Perceived susceptibility to prolonged sitting was assessed with 2 items (e.g. “*My daily sitting time is more compared to what is recommended.*”; *r* = .72) [23, 24].

The Individual Work Performance Questionnaire (seldom = 0 to always = 5) was used to assess task and contextual performance. Task performance (5 items; *ω* = .72) refers to the ability to perform the tasks that are essential, e.g. “*During the last week, I was able to perform my work well with minimal time and effort*”. Contextual performance (9 items; *ω* = .57) refers to the organizational, social, or psychological factors that are required for adequate functioning at work, e.g. “*I took on extra responsibilities*.” [25]*.* Furthermore, we used the Perceived Stress Scale (10 items; 1 = *never*, 5 = *very often*; e.g. “*How often have you felt nervous and ‘stressed’?*”; *ω* = .89) [26, 27] and the bodily pain (2 items; e.g. “*How much bodily pain have you had?*”; *r* = .85), emotional well-being (5 items; e.g. “*How much of the time have you been a happy person?*”; *ω* = .83), and vitality (4 items; e.g. “*How much of the time did you have a lot of energy?*”; *ω* = .86) sub-scales of the SF-36 [28].

The VitaBit tool measures SB, standing, and physical activity, but does not distinguish between different intensities of physical activity (i.e. light versus moderate-to-vigorous activity). Hence, we additionally assessed light and MVPA with the German version of the international physical activity questionnaire short form (max. 6 items; excluding SB; e.g. “*During the last 7 days, on how many days did you do vigorous physical activities like heavy lifting, digging, heavy construction, or climbing stairs as part of your work?*”) [29].

### Data Analyses

To clean and analyse the data, we used R version 4.0.2. We inspected the data using descriptive univariate analyses, and we visualized them with histograms and QQ plots to check for normality. We reported normally distributed variables as means and standard deviations (SD), non-normally distributed variables as medians and inter-quartile ranges (IQR), and categorical variables as absolute numbers and percentages. SB was represented as proportion of the entire waking day (i.e. when the device was worn) by applying a compositional data approach (CoDA) (i.e. ${z1}_{sitting}=\sqrt{2/3 } \;\mathrm{ln}(Sitting\%/\sqrt{Standing\% \times Activity\%} ))$ [30] and as sum of the squared sitting bouts (SSSB) ($SSSB=\sum_{0}^{n}{SitBout}_{i}^{2}$) [13]. We used only those days where a participant had collected at least 8 h of physical behaviour data (i.e. the sum of minutes measured as sitting, standing, or active) [, 15, 31], and we excluded holidays from the analyses. The Mahalanobis distance method was used to detect and exclude outliers [32].

To calculate the within-subjects improvements of SB, QoL, and psychosocial determinants, we only used calendar weeks, in which baseline data (i.e. of other participants still being in their baseline week, i.e. control condition) were available. These baseline data were used to centre the outcome variables to control for seasonal trends. The within-subjects improvements of the variables (in %) that were collected with surveys were calculated as follows. If lower values were considered healthier, such as in perceived stress, survey 2 was subtracted from survey 1, else survey 1 was subtracted from survey 2. We then divided by survey 1 to retrieve the percentual improvement. If the survey was filled out 3 times, additionally, the same calculation was performed with surveys 2 and 3. We then calculated a survey-to-survey improvement by using the mean of the two results. For SB, we took the averages for calendar weeks and calculated the week-to-week improvements.

Linear regression models were used with ordinary least squares, if residuals were normally distributed, else with percentage least squares [33], to explore potential moderators of effectiveness. Thereby, participant characteristics (e.g. gender, age, company industry, BMI), baseline physical behaviours (e.g. SB, MVPA), baseline QoL (e.g. perceived stress, vitality), and intervention perception (e.g. understandability, acceptance of the intervention) were regressed on within-subjects improvements (i.e. difference scores after centring around calendar week means [34]) in psychosocial determinants (e.g. attitude, perceived social norms), on performance objectives (e.g. average registering, monitoring), on improvements in SB, and in QoL. For testing statistical significance (two-sided), we used an alpha of 0.05, which we corrected by the help of the Benjamini–Hochberg procedure [35, 36].

To gather insights into potential ceiling effects, additional post hoc analyses were done with a sub-group of participants who did not score as high in relevant determinants and quality of life variables. The seven variables that were found to be associated with the effectiveness of UPcomplish were used to create this sub-group: only participants who scored below the median in at least four (i.e. the majority) of these seven variables were included in this sub-group (*n* = 51). We calculated pairwise Pearson’s correlations between all variables of the 4 parts of the logic model of the intervention (i.e. psychosocial determinants, performance objectives, SB, and QoL). A positive improvement can be interpreted as a beneficial intra-individual week-to-week (as in SB) or as a measurement-to-measurement (as in QoL) development. Week-to-week SB improvement was calculated as proportional improvement in %, measurement-to-measurement improvement of the survey variables was calculated as average absolute improvement. For this analysis, we did not centre the variables around calendar week means, because of the lower number of participants and available baseline data in the concerning calendar weeks.

## Results

### Participant Characteristics

Table 1 presents the descriptive characteristics of the sample at baseline. Among the participants who agreed to participate, 143 (47 males) participants with a median age of 42.0 (IQR = 21.5) years and a mean BMI of 23.4 (SD = 5.2) kg/m^2^ created a VitaBit account. Males had a higher (*p* < .01) BMI than females. At baseline, most participants indicated that their work tasks encompassed mainly computer and/or desk work. The majority had a full-time position and a usual work week of 5 workdays.

**Table 1:** Descriptive characteristics of participants at baseline

	**Female**	**Male**	**Total**
	*n* = 97	*n* = 47	*n* = 143
**Participant characteristics**			
Age (years), median (IQR)	41.0 (20.5)	44.0 (19.5)	42.0 (21.5)
Anthropometrics, mean (SD)			
Height (cm)	168.6 (6.9)	180.5 (6.7)	172.4 (8.8)
Weight (kg)	65.0 (13.0)	82.0 (16.0)	70.0 (22.5)
BMI (kg/m^2^)	22.3 (5.1)	25.6 (4.8)	23.4 (5.2)
Job-related variables			
Main work tasks, *n* (%^a^)			
* Computer and desk work*	26 (26.8)	9 (19.1)	35 (24.5)
* Computer work*	37 (38.1)	23 (48.9)	60 (42.0)
* Desk work*	21 (21.6)	7 (14.9)	28 (19.6)
Work status, *n* (%^a^)			
* Full-time*	65 (67.0)	39 (83.0)	104 (72.7)
* Part-time*	20 (20.6)	1 (2.1)	21 (14.7)
Workdays per week, *n* (%^a^)			
* 4 workdays*	7 (7.2)	2 (4.3)	9 (6.3)
* 5 workdays*	76 (78.4)	37 (78.7)	113 (79.0)
* 6 workdays*	0 (0.0)	3 (6.4)	3 (2.1)
**Psychosocial determinants**			
Attitude, median (IQR)	4.3 (0.8)	4.2 (0.5)	4.2 (0.7)
Perceived social support, mean (SD)	3.4 (0.9)	3.3 (0.9)	3.4 (0.9)
PBC, median (IQR)	4.0 (1.0)	4.0 (0.9)	4.0 (1.0)
Perceived susceptibility, median (IQR)	5.0 (1.0)	5.0 (1.0)	5.0 (1.0)
Intention, mean (SD)	3.5 (0.8)	3.7 (0.9)	3.6 (0.9)
**Physical behaviou**r			
Wear time (min d^−1^), mean (SD)	835.7 (102.0)	797.8 (115.2)	823.4 (107.5)
SB (min d^−1^), median (IQR)	504.4 (96.5)	522.3 (92.7)	510.2 (95.3)
SB compositional geometric mean^c^, log-ratio variances standing, walking	62.3 (0.3, 0.2)	67.7 (0.2, 0.2)	64.3 (0.3, 0.2)
Standing (min d^−1^), median (IQR)	224.8 (129.7)	161.3 (73.2)	199.6 (102.8)
Standing compositional geometric mean^c^, log-ratio variances sitting, walking	27.2 (0.3, 0.2)	19.4 (0.2, 0.1)	24.5 (0.3, 0.3)
Activity (min d^−1^), median (IQR)	83.9 (45.6)	105.2 (37.8)	91.7 (45.7)
Activity compositional geometric mean^c^, log-ratio variances sitting, standing	10.5 (0.2, 0.2)	12.9 (0.2, 0.1)	11.3 (0.2, 0.3)
**Quality of life**			
Task performance, mean (SD)	3.6 (0.5)	3.4 (0.7)	3.6 (0.6)
Contextual performance, mean (SD)	3.3 (0.6)	3.3 (0.6)	3.3 (0.6)
Perceived stress, mean (SD)	15.0 (9.5)	16.0 (10.0)	15.0 (10.0)
Perceived pain, mean (SD)	77.5 (32.5)	87.5 (32.5)	77.5 (32.5)
Vitality, mean (SD)	54.5 (18.2)	54.3 (20.3)	54.4 (18.8)
Emotional well-being, mean (SD)	72.0 (18.0)	80.0 (20.0)	76.0 (20.0)

*SD* standard deviation, *IQR* interquartile range, *min d*^*−1*^ minutes per day, *%/d* proportion of the day

^a^Proportion of the sample. If not all participants filled out the survey, the percentages do not add up to 100%

^b^Estimates of physical behaviours are estimated via VitaBit accelerometery

^c^The percentage of the day is the estimated proportion of wearing-minutes spent in each activity level

The psychosocial determinants (range 1 to 5) regarding sitting ranged from a mean of 3.4 (SD = 0.9) for perceived social support to a median of 5.0 (IQR = 1.0) for perceived susceptibility. At baseline, the participants wore their VitaBit device on average for 823.4 (SD = 107.5) min per day, of which the device measured a median of 510.2 (IQR = 95.3) SB minutes, 199.6 (IQR = 102.8) standing minutes, and 91.7 (IQR = 45.7) activity minutes. Females collected more (*p* < .001) standing time than males, while males collected more (*p* < 0.01) activity time than females. Performance at baseline was on average 3.3 (SD = 0.6) for task and 3.6 (SD = 0.6) for contextual performance (1 to 5). On average, perceived stress (0 = no stress, 40 = high stress) was 15.0 (SD = 10.0), perceived pain (0 = much pain, 100 = no pain) was 77.5 (SD = 32.5), and vitality and emotional well-being (both 0 = low, 100 = high) were 54.4 (SD = 18.8) and 76.0 (SD = 20.0), respectively.

### Variables Affecting Improvements in Psychosocial Determinants

Table 2 presents the results of the regression models exploring moderators affecting improvements in psychosocial determinants. After Benjamini–Hochberg corrections, higher baseline intentions were associated with significantly less improvement in intention during participation in the intervention. None of the other improvements in determinants was related to participant characteristics, job-related variables, baseline behaviours, or how the intervention messages were perceived.

**Table 2:** Linear regression models for the effects of participant characteristics, baseline variables, and intervention perception on improvements in psychosocial determinants

	*n*	*Improvement attitude*		*Improvement PBC*		*Improvement PSS*		*Improvement PS*		*Improvement intention*	
		*β (SE)*	95% CI	*β (SE)*	95% CI	*β (SE)*	95% CI	*β (SE)*	95% CI	*β (SE)*	95% CI
Gender	56	− 0.05 (0.18)	− 0.41, 0.31	− 0.08 (0.22)	− 0.52, 0.37	− 0.28 (0.25)	− 0.78, 0.22	− 0.32 (0.27)	− 0.87, 0.23	− 0.26 (0.26)	− 0.78, 0.26
Age (years)	54	0 (0.01)	− 0.02, 0.01	− 0.01 (0.01)	− 0.03, 0.01	0 (0.01)	− 0.02, 0.02	0.01 (0.01)	− 0.01, 0.04	0 (0.01)	− 0.02, 0.02
BMI (kg/m^2^)	42	0.02 (0.02)	− 0.01, 0.06	0.01 (0.02)	− 0.03, 0.05	− 0.03 (0.02)	− 0.08, 0.02	− 0.02 (0.03)	− 0.07, 0.04	0.05 (0.02)	0, 0.09
Work status	55	− 0.07 (0.22)	− 0.5, 0.37	0.12 (0.27)	− 0.42, 0.66	− 0.09 (0.31)	− 0.71, 0.53	− 0.67 (0.32)	− 1.32, − 0.03	− 0.37 (0.31)	− 0.99, 0.25
Computer work (%/day)	56	0 (0)	− 0.01, 0.01	− 0.01 (0.01)	− 0.02, 0.01	0 (0.01)	− 0.01, 0.01	0 (0.01)	− 0.02, 0.01	− 0.01 (0.01)	− 0.02, 0.01
Desk work (%/day)	52	0 (0)	0, 0.01	0 (0)	− 0.01, 0.01	0 (0)	− 0.01, 0	0 (0)	− 0.01, 0.01	0.01 (0)	0, 0.01
Meetings (%/day)	53	0 (0.01)	− 0.02, 0.02	0.01 (0.02)	− 0.02, 0.04	− 0.01 (0.02)	− 0.05, 0.02	− 0.01 (0.02)	− 0.05, 0.03	0.02 (0.02)	− 0.01, 0.06
Phone calls (%/day)	56	0.02 (0.01)	0, 0.03	0.01 (0.01)	0, 0.03	− 0.02 (0.01)	− 0.04, 0	0.01 (0.01)	− 0.01, 0.03	0 (0.01)	− 0.02, 0.02
Travels/customers (%/day)	32	0 (0.01)	− 0.03, 0.03	− 0.01 (0.02)	− 0.05, 0.03	− 0.01 (0.03)	− 0.06, 0.04	0.03 (0.02)	− 0.02, 0.08	0.02 (0.02)	− 0.02, 0.07
Attitude	56	− 0.29 (0.16)	− 0.61, 0.03	− 0.09 (0.21)	− 0.51, 0.32	− 0.09 (0.23)	− 0.56, 0.38	0.02 (0.26)	− − 0.5, 0.53	− 0.16 (0.24)	− 0.65, 0.33
PBC	56	− 0.09 (0.13)	− 0.34, 0.16	− 0.23 (0.15)	− 0.54, 0.08	− 0.12 (0.18)	− 0.48, 0.23	0.07 (0.2)	− 0.33, 0.46	− 0.06 (0.19)	− 0.43, 0.31
Perceived social support	56	0.06 (0.1)	− 0.14, 0.26	0.06 (0.13)	− 0.2, 0.31	** − 0.4 (0.13)**	** − 0.66, − 0.13**	0.1 (0.16)	− 0.22, 0.41	0.12 (0.15)	− 0.17, 0.42
Perceived susceptibility	56	− 0.01 (0.12)	− 0.25, 0.23	− 0.02 (0.15)	− 0.32, 0.27	0.04 (0.17)	− 0.3, 0.38	** − 0.49 (0.17)**	** − 0.84, − 0.14**	0.06 (0.18)	− 0.3, 0.41
Intention	56	0.01 (0.11)	− 0.21, 0.22	0.02 (0.14)	− 0.25, 0.29	− 0.14 (0.15)	− 0.45, 0.16	− 0.28 (0.16)	− 0.61, 0.05	** − 0.52 (0.14)**	** − 0.81, − 0.23**
MVPA	49	0 (0)	0, 0	0 (0)	0, 0	0 (0)	0, 0	0 (0)	0, 0	0 (0)	0, 0
z1_SB	56	− 0.07 (0.23)	− 0.52, 0.38	0.12 (0.28)	− 0.45, 0.68	0.19 (0.32)	− 0.45, 0.83	− 0.25 (0.35)	− 0.95, 0.45	0.07 (0.33)	− 0.6, 0.73
SSSB	56	0 (0)	0, 0	0 (0)	0, 0	0 (0)	0, 0	0 (0)	0, 0	0 (0)	0, 0
Task performance	55	− 0.08 (0.13)	− 0.35, 0.18	− 0.16 (0.16)	− 0.47, 0.15	0.02 (0.18)	− 0.35, 0.39	− 0.06 (0.21)	− 0.48, 0.35	0 (0.2)	− 0.39, 0.4
Contextual performance	56	− 0.07 (0.16)	− 0.39, 0.25	− 0.17 (0.2)	− 0.57, 0.22	− 0.05 (0.22)	− 0.5, 0.4	− 0.07 (0.25)	− 0.56, 0.42	− 0.06 (0.23)	− 0.53, 0.41
Perceived stress	56	0.01 (0.01)	− 0.02, 0.04	0 (0.02)	− 0.03, 0.04	0.03 (0.02)	− 0.01, 0.07	0 (0.02)	− 0.04, 0.05	0.02 (0.02)	− 0.02, 0.07
Perceived pain	56	− 0.01 (0)	− 0.01, 0	− 0.01 (0)	− 0.01, 0	0.01 (0.01)	0, 0.02	− 0.01 (0.01)	− 0.02, 0	− 0.01 (0.01)	− 0.02, 0
Vitality	56	− 0.01 (0)	− 0.01, 0	0 (0.01)	− 0.01, 0.01	− 0.01 (0.01)	− 0.02, 0	0.01 (0.01)	− 0.01, 0.02	− 0.01 (0.01)	− 0.02, 0.01
Emotional well-being	56	− 0.01 (0.01)	− 0.02, 0	− 0.01 (0.01)	− 0.02, 0.01	− 0.02 (0.01)	− 0.04, 0	0 (0.01)	− 0.02, 0.02	− 0.01 (0.01)	− 0.03, 0.01
Acceptability	42	0.12 (0.15)	− 0.18, 0.42	− 0.06 (0.17)	− 0.4, 0.27	− 0.11 (0.17)	− 0.45, 0.24	0.19 (0.22)	− 0.26, 0.64	0.37 (0.2)	− 0.04, 0.78
Understandability	42	0 (0.18)	− 0.37, 0.36	− 0.12 (0.2)	− 0.52, 0.29	− 0.12 (0.21)	− 0.54, 0.3	0.08 (0.27)	− 0.47, 0.64	0.05 (0.26)	− 0.47, 0.57
Message processing	42	− 0.05 (0.09)	− 0.24, 0.14	− 0.05 (0.1)	− 0.27, 0.16	0.06 (0.11)	− 0.16, 0.28	0.14 (0.14)	− 0.14, 0.42	− 0.15 (0.13)	− 0.42, 0.11

Cohen’s [52] ***f***^**2**^ ≥ **0.15** (medium) and ***f***^**2**^ ≥ 0.35* (large) effect sizes

*SE* standard error, *IQR* interquartile range, *min d*^*−1*^ min per day, *%/d* proportion of the day

### Variables Affecting the Performance Objectives

Table 3 presents the results of the regression models exploring factors being associated with performance objectives. None of the performance objectives was associated with participant characteristics, job-related variables, baseline behaviours, or how the intervention messages were perceived.

**Table 3:** Linear regression models for the effects of participant characteristics, baseline variables, and intervention perception on average performance objectives

	*n*	*Monitoring*		*n*	*Registering*		*n*	*Engaging*	
		*β (SE)*	95% CI		*β (SE)*	95% CI		*β (SE)*	95% CI
Gender	139	0.06 (0.24)	− 0.41, 0.52	142	0.13 (0.2)	− 0.27, 0.53	131	0.39 (4.66)	− 8.84, 9.62
Age (years)	132	− 0.02 (0.01)	− 0.04, 0	135	0.02 (0.01)	0.01, 0.04	124	0.37 (0.19)	− 0.01, 0.75
BMI (kg/m^2^)	106	0.02 (0.02)	− 0.03, 0.07	108	0.01 (0.02)	− 0.03, 0.05	99	− 0.1 (0.46)	− 1.01, 0.81
Work status	123	0.35 (0.3)	− 0.25, 0.94	126	− 0.67 (0.25)	− 1.16, − 0.18	119	1.26 (5.67)	− 9.96, 12.49
Computer work (%/day)	124	0 (0.01)	− 0.01, 0.02	127	− 0.01 (0)	− 0.02, 0	120	− 0.08 (0.1)	− 0.28, 0.13
Desk work (%/day)	117	0 (0)	− 0.01, 0.01	120	− 0.01 (0)	− 0.01, 0	114	− 0.09 (0.06)	− 0.21, 0.04
Meetings (%/day)	118	− 0.01 (0.01)	− 0.03, 0.02	120	− 0.03 (0.01)	− 0.05, − 0.01	114	0.09 (0.24)	− 0.39, 0.57
Phone calls (%/day)	121	0.01 (0.01)	− 0.01, 0.02	124	− 0.01 (0.01)	− 0.03, 0	117	0.04 (0.15)	− 0.26, 0.34
Travels/customers (%/day)	59	0 (0.02)	− 0.03, 0.03	60	− 0.01 (0.01)	− 0.03, 0.02	55	− 0.31 (0.27)	− 0.85, 0.23
Attitude	124	− 0.16 (0.23)	− 0.62, 0.3	127	0.11 (0.2)	− 0.28, 0.49	120	− 5.6 (4.35)	− 14.23, 3.02
PBC	124	− 0.32 (0.17)	− 0.65, 0.01	127	0.33 (0.14)	0.06, 0.6	120	− 2.99 (3.18)	− 9.29, 3.3
Perceived social support	124	0.11 (0.13)	− 0.14, 0.37	127	0.02 (0.11)	− 0.2, 0.23	120	− 1.64 (2.43)	− 6.44, 3.17
Perceived susceptibility	124	0.16 (0.15)	− 0.14, 0.46	127	− 0.09 (0.13)	− 0.35, 0.16	120	− 2.74 (2.89)	− 8.47, 2.99
Intention	124	− 0.11 (0.14)	− 0.38, 0.16	127	0.19 (0.11)	− 0.03, 0.42	120	0.76 (2.63)	− 4.44, 5.97
MVPA	116	0 (0)	0, 0	119	0 (0)	0, 0	114	0 (0)	0, 0
z1_SB	139	0.63 (0.3)	0.03, 1.22	142	− 0.45 (0.26)	− 0.96, 0.07	131	− 1.32 (6.18)	− 13.54, 10.91
SSSB	139	0 (0)	0, 0	142	0 (0)	0, 0	131	0 (0)	0, 0
Task performance	123	0.04 (0.2)	− 0.35, 0.43	123	− 0.15 (0.16)	− 0.47, 0.16	119	− 0.79 (3.7)	− 8.12, 6.53
Contextual performance	124	0.39 (0.2)	− 0.01, 0.79	124	− 0.07 (0.17)	− 0.4, 0.27	120	− 3.15 (3.88)	− 10.84, 4.53
Perceived stress	124	0.05 (0.02)	0.01, 0.08	124	− 0.01 (0.02)	− 0.04, 0.02	120	− 0.21 (0.36)	− 0.93, 0.5
Perceived pain	122	− 0.01 (0.01)	− 0.02, 0	122	0.01 (0)	0, 0.01	118	0.09 (0.1)	− 0.11, 0.28
Vitality	124	− 0.01 (0.01)	− 0.02, 0.01	124	0.01 (0.01)	0, 0.02	120	0.07 (0.12)	− 0.16, 0.3
Emotional well-being	124	− 0.02 (0.01)	− 0.03, 0	124	0 (0.01)	− 0.01, 0.02	120	0.04 (0.16)	− 0.27, 0.35
Acceptability	62	− 0.13 (0.23)	− 0.59, 0.32	62	0.09 (0.17)	− 0.24, 0.43	60	1.87 (3.64)	− 5.43, 9.16
Understandability	62	0 (0.28)	− 0.56, 0.56	62	− 0.03 (0.21)	− 0.44, 0.38	60	3.79 (4.3)	− 4.81, 12.38
Message processing	62	− 0.06 (0.15)	− 0.36, 0.24	62	0.12 (0.11)	− 0.1, 0.34	60	4.67 (2.28)	0.12, 9.22

Cohen’s [52] ***f***^**2**^ ≥ **0.15** (medium) and ***f***^**2**^ ≥ 0.35* (large) effect sizes

*SE* standard error, *IQR* interquartile range, *min d*^−*1*^ min per day, *%/d* proportion of the day

### Variables Affecting Improvements in Sedentary Behaviour

Table 4 presents the results of the regression models exploring variables being associated with SB improvement. SB improvement was not found to be associated with participant characteristics, job-related variables, baseline behaviours, or how the intervention messages were perceived.

**Table 4:** Linear regression models for the effects of participant characteristics, baseline variables, and intervention perception on improvements in SB

	*n*	*SB CoDA*		*n*	*SSSB*	
		*β (SE)*	95% CI		*β (SE)*	95% CI
Gender	116	− 1.27 (3.48)	− 8.17, 5.64	120	− 55.49 (51.57)	− 157.62, 46.63
Age (years)	109	− 3.22 (8.67)	− 20.47, 14.02	115	− 4.34 (2.12)	− 8.54, − 0.15
BMI (kg/m^2^)	87	− 17.15 (103.2)	− 221.68, 187.37	92	− 6.33 (5.72)	− 17.7, 5.04
Work status	112	− 2.37 (1.89)	− 6.11, 1.38	116	− 2.85 (65.95)	− 133.5, 127.8
Computer work (%/day)	113	− 0.58 (1.2)	− 2.95, 1.8	117	− 1.04 (1.2)	− 3.41, 1.34
Desk work (%/day)	106	7.41 (4.24)	− 1.01, 15.82	110	− 0.63 (0.71)	− 2.05, 0.78
Meetings (%/day)	107	− 3.14 (2.66)	− 8.41, 2.12	110	− 5.36 (2.64)	− 10.6, − 0.13
Phone calls (%/day)	111	− 12.47 (4.61)	− 21.74, − 3.21	114	− 1.52 (1.7)	− 4.89, 1.85
Travels/customers (%/day)	53	45.5 (79.9)	− 112.84, 203.83	54	− 4.04 (2.77)	− 9.6, 1.52
Attitude	113	65.64 (56.18)	− 45.68, 176.96	117	62.24 (50.45)	− 37.69, 162.17
PBC	113	92.38 (42.36)	8.44, 176.32	117	34.47 (36.07)	− 36.98, 105.92
Perceived social support	113	− 20.89 (51.07)	− 122.08, 80.3	117	25.52 (27.19)	− 28.33, 79.37
Perceived susceptibility	113	54.69 (46.11)	− 36.69, 146.06	117	11.76 (32.2)	− 52.02, 75.53
Intention	113	0.03 (0.02)	− 0.01, 0.07	117	− 18.63 (28.31)	− 74.71, 37.44
MVPA	106	− 91.78 (104.16)	− 298.11, 114.55	109	0.02 (0.01)	0, 0.04
z1_SB	116	− 0.01 (0)	− 0.02, 0	120	54.5 (65.23)	− 74.68, 183.68
SSSB	116	− 61.55 (65.96)	− 192.29, 69.18	120	0 (0)	− 0.01, 0
Task performance	110	38.31 (69.06)	− 98.57, 175.18	113	18.64 (39.85)	− 60.33, 97.61
Contextual performance	111	− 4.05 (6.57)	− 17.06, 8.96	114	− 112.04 (42.48)	− 196.21, − 27.86
Perceived stress	111	2.29 (1.72)	− 1.13, 5.71	114	1.22 (3.99)	− 6.7, 9.13
Perceived pain	109	1.12 (2.14)	− 3.12, 5.37	113	2.04 (1.06)	− 0.06, 4.13
Vitality	111	3.1 (2.81)	− 2.46, 8.66	114	− 2.43 (1.28)	− 4.96, 0.11
Emotional well-being	111	60.09 (74.85)	− 89.85, 210.04	114	− 1.39 (1.72)	− 4.8, 2.03
Acceptability	58	114.61 (90.32)	− 66.32, 295.55	58	22.72 (52.17)	− 81.79, 127.24
Understandability	58	1.55 (48.09)	− 94.78, 97.89	58	30.88 (61.51)	− 92.34, 154.1
Message processing	58	− 1.27 (3.48)	− 8.17, 5.64	58	28.47 (33.33)	− 38.31, 95.24

*SE* standard error, *IQR* interquartile range, *min d*^*−1*^ minutes per day, *%/d* proportion of the day

Cohen’s [52] ***f***^**2**^ ≥ **0.15** (medium) and ***f***^**2**^ ≥ 0.35* (large) effect sizes

### Variables Affecting Improvements in Quality of Life

After the Benjamini–Hochberg correction, most of the QoL improvements were associated with their own baseline values (see Tables 5 and 6). Higher baseline task performance was associated with fewer improvements in task performance, higher baseline stress with more improvement in perceived stress, higher baseline vitality with less improvement in vitality, and higher baseline emotional well-being with less improvement in emotional well-being. Furthermore, lower baseline stress and higher baseline emotional well-being were associated with more improvement in contextual performance. Finally, higher baseline attitude and PBC were associated with fewer improvements in emotional well-being.

**Table 5:** Linear regression models for the effects of participant characteristics, baseline variables, and intervention perception on improvements in quality of life

	*n*	*Task performance*		*Contextual performance*		*Perceived stress*	
		*β (SE)*	95% CI	*β (SE)*	95% CI	*β (SE)*	95% CI
Gender	56	− 0.22 (0.16)	− 0.54, 0.09	0.01 (0.19)	− 0.38, 0.4	− 1.44 (1.55)	− 4.54, 1.66
Age (years)	54	0 (0.01)	− 0.01, 0.01	0 (0.01)	− 0.01, 0.02	0.05 (0.07)	− 0.08, 0.19
BMI (kg/m^2^)	42	− 0.01 (0.02)	− 0.04, 0.02	0.01 (0.02)	− 0.02, 0.05	0.04 (0.13)	− 0.23, 0.31
Work status	55	0.04 (0.19)	− 0.35, 0.42	− 0.24 (0.23)	− 0.71, 0.23	0.58 (1.9)	− 3.24, 4.39
Computer work	56	0 (0)	− 0.01, 0	0 (0)	− 0.01, 0.01	− 0.01 (0.04)	− 0.09, 0.07
Desk work	52	0 (0)	0, 0.01	0 (0)	− 0.01, 0.01	0.05 (0.02)	0, 0.1
Meetings	53	0.01 (0.01)	− 0.01, 0.03	0 (0.01)	− 0.03, 0.02	**0.28 (0.1)**	**0.08, 0.48**
Phone calls	56	0.01 (0.01)	− 0.01, 0.02	0 (0.01)	− 0.02, 0.02	0.1 (0.06)	− 0.02, 0.23
Travels/customers	32	0 (0.01)	− 0.03, 0.02	− 0.02 (0.02)	− 0.05, 0.02	0.14 (0.13)	− 0.13, 0.4
Attitude	56	− 0.15 (0.15)	− 0.45, 0.14	0.45 (0.17)	0.11, 0.79	− 1.72 (1.42)	− 4.57, 1.14
PBC	56	− 0.13 (0.11)	− 0.35, 0.1	0.33 (0.13)	0.07, 0.59	− 2.76 (1.04)	− 4.84, − 0.68
PSS	56	− 0.09 (0.09)	− 0.27, 0.09	0.27 (0.1)	0.06, 0.47	− 1.44 (0.86)	− 3.16, 0.28
PS	56	0.15 (0.11)	− 0.07, 0.37	0.08 (0.13)	− 0.18, 0.34	− 2.02 (1.01)	− 4.04, 0
Intention	56	− 0.04 (0.1)	− 0.23, 0.15	− 0.03 (0.12)	− 0.26, 0.21	− 0.94 (0.94)	− 2.82, 0.94
MVPA	49	0 (0)	0, 0	0 (0)	0, 0	0 (0)	0, 0
z1_SB	56	− 0.43 (0.2)	− 0.83, − 0.03	0.02 (0.25)	− 0.47, 0.51	− 4.96 (1.86)	− 8.69, − 1.24
SSSB	56	0 (0)	0, 0	0 (0)	0, 0	0 (0)	0, 0
TP	55	− 0.45 (0.1)*	− 0.65, − 0.25	0.23 (0.14)	− 0.05, 0.51	− 2.08 (1.04)	− 4.15, 0
CP	56	− 0.16 (0.15)	− 0.46, 0.13	− 0.1 (0.17)	− 0.44, 0.25	− 0.79 (1.38)	− 3.56, 1.98
Perceived stress	56	0.01 (0.01)	− 0.02, 0.03	** − 0.05 (0.01)**	** − 0.08, − 0.02**	**0.41 (0.11)**	**0.18, 0.63**
Perceived pain	56	0 (0)	− 0.01, 0.01	0 (0)	− 0.01, 0	− 0.07 (0.03)	− 0.13, − 0.01
Vitality	56	0 (0)	− 0.01, 0.01	0 (0)	− 0.01, 0.01	− 0.01 (0.04)	− 0.09, 0.06
EWB	56	0 (0.01)	− 0.01, 0.01	**0.02 (0.01)**	**0.01, 0.03**	− 0.13 (0.05)	− 0.23, − 0.03
Accept	42	− 0.15 (0.12)	− 0.4, 0.1	0.06 (0.14)	− 0.23, 0.35	− 0.15 (1.26)	− 2.69, 2.39
Understand	42	− 0.17 (0.15)	− 0.47, 0.13	0.05 (0.17)	− 0.3, 0.4	0.49 (1.52)	− 2.59, 3.57
Message processing	42	0.04 (0.08)	− 0.12, 0.21	− 0.02 (0.09)	− 0.2, 0.16	− 0.06 (0.8)	− 1.67, 1.55

*SE* standard error, *IQR* interquartile range, *min d*^*−1*^ minutes per day, *%/d* proportion of the day

Cohen’s [52] ***f***^**2**^ ≥ **0.15** (medium) and ***f***^**2**^ ≥ 0.35* (large) effect sizes

**Table 6:** Linear regression models for the effects of participant characteristics, baseline variables, and intervention perception on improvements in quality of life

	*n*	*Pain*		*Vitality*		*Emotional well-being*	
		*β (SE)*	95% CI	*β (SE)*	95% CI	*β (SE)*	95% CI
Gender	56	− 13.25 (5.9)	− 25.08, − 1.42	0.24 (4.25)	− 8.28, 8.76	− 5.26 (4.59)	− 14.47, 3.96
Age (years)	54	0.16 (0.26)	− 0.35, 0.68	0.02 (0.16)	− 0.3, 0.34	0.18 (0.19)	− 0.21, 0.57
BMI (kg/m^2^)	42	0.46 (0.61)	− 0.77, 1.68	0.32 (0.35)	− 0.39, 1.04	− 0.44 (0.4)	− 1.25, 0.38
Work status	55	− 3.47 (7.49)	− 18.5, 11.56	− 0.7 (5.18)	− 11.09, 9.69	− 6.25 (5.6)	− 17.48, 4.99
Computer work	56	0.02 (0.15)	− 0.28, 0.33	− 0.15 (0.1)	− 0.36, 0.06	− 0.09 (0.11)	− 0.32, 0.14
Desk work	52	0.14 (0.1)	− 0.05, 0.34	− 0.04 (0.07)	− 0.17, 0.1	− 0.07 (0.07)	− 0.22, 0.07
Meetings	53	0.5 (0.43)	− 0.37, 1.36	0.31 (0.29)	− 0.27, 0.89	0.55 (0.29)	− 0.03, 1.13
Phone calls	56	0.05 (0.25)	− 0.46, 0.55	− 0.28 (0.17)	− 0.62, 0.06	0.14 (0.19)	− 0.24, 0.52
Travels/customers	32	** − 1.1 (0.52)**	** − 2.17, − 0.03**	0.39 (0.36)	− 0.35, 1.12	0.22 (0.36)	− 0.52, 0.96
Attitude	56	− 6.89 (5.63)	− 18.19, 4.41	− 2.53 (3.92)	− 10.39, 5.32	− 12.92 (3.93)	− 20.8, − 5.04
PBC	56	− 6.74 (4.27)	− 15.31, 1.83	− 6.49 (2.88)	− 12.26, − 0.72	** − 9.27 (3.04)**	** − 15.37, − 3.16**
PSS	56	− 1.06 (3.49)	− 8.04, 5.93	− 3.29 (2.36)	− 8.03, 1.44	− 4.01 (2.57)	− 9.17, 1.15
PS	56	− 2.29 (4.12)	− 10.54, 5.96	2.81 (2.82)	− 2.84, 8.46	− 4.68 (3.05)	− 10.79, 1.42
Intention	56	− 0.89 (3.75)	− 8.39, 6.62	− 1.21 (2.58)	− 6.38, 3.95	0.81 (2.82)	− 4.84, 6.47
MVPA	49	0 (0)	− 0.01, 0	0 (0)	0, 0	0 (0)	0, 0
z1_SB	56	− 11.84 (7.65)	− 27.18, 3.5	− 0.1 (5.38)	− 10.89, 10.7	− 8.61 (5.77)	− 20.19, 2.96
SSSB	56	0 (0)	0, 0	0 (0)	0, 0	0 (0)	0, 0
TP	55	− 0.43 (4.47)	− 9.38, 8.53	− 3.73 (3.12)	− 9.99, 2.52	− 4.41 (3.34)	− 11.11, 2.29
CP	56	− 4.09 (5.46)	− 15.03, 6.86	− 1.83 (3.77)	− 9.39, 5.73	− 3.31 (4.11)	− 11.55, 4.94
Perceived stress	56	0.5 (0.49)	− 0.48, 1.48	0.46 (0.33)	− 0.21, 1.13	0.83 (0.35)	0.12, 1.54
Perceived pain	56	− 0.32 (0.12)	− 0.56, − 0.09	− 0.12 (0.08)	− 0.29, 0.05	− 0.15 (0.09)	− 0.34, 0.03
Vitality	56	0.09 (0.15)	− 0.21, 0.4	** − 0.33 (0.1)**	** − 0.52, − 0.14**	− 0.11 (0.11)	− 0.34, 0.12
EWB	56	− 0.01 (0.21)	− 0.44, 0.41	− 0.26 (0.14)	− 0.54, 0.02	− 0.48 (0.15)	− 0.77, − 0.19
Accept	42	3.87 (4.7)	− 5.63, 13.37	0.43 (3.61)	− 6.87, 7.73	1 (3.66)	− 6.4, 8.4
Understand	42	− 4.94 (5.7)	− 16.46, 6.58	3.09 (4.36)	− 5.71, 11.9	− 0.85 (4.45)	− 9.84, 8.14
Message processing	42	− 3.43 (2.95)	− 9.39, 2.53	− 2.26 (2.26)	− 6.82, 2.31	1.56 (2.31)	− 3.1, 6.22

Cohen’s [52] ***f***^**2**^ ≥ **0.15** (medium) and ***f***^**2**^ ≥ 0.35* (large) effect sizes

*SE* standard error, *IQR* interquartile range, *min d*^*−1*^ minutes per day, *%/d* proportion of the day

### Post HocAnalyses: Can Subjects Scoring Low on Relevant Determinants and Quality of Life Profit?

**Fig. 5:**
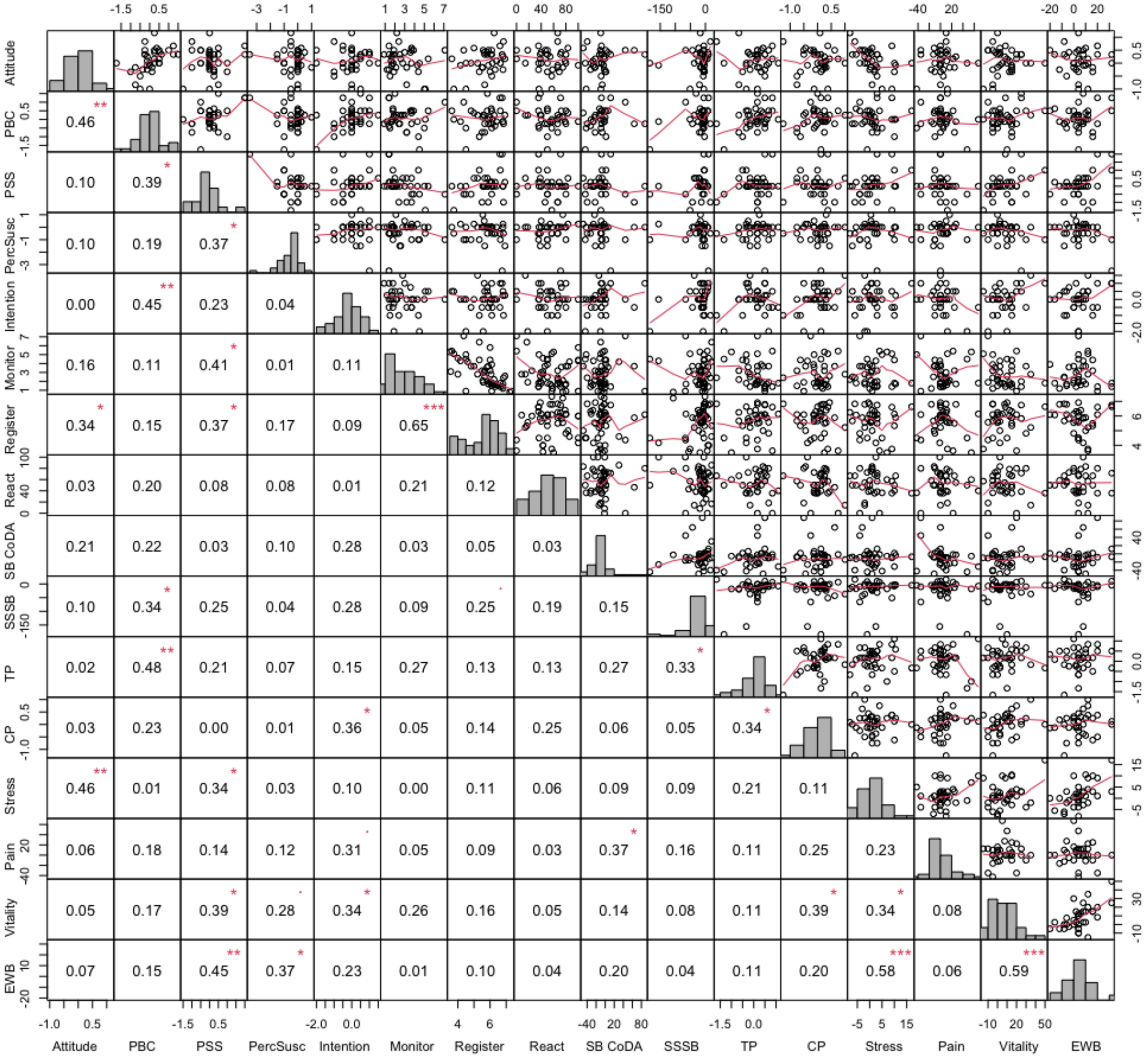
Pearson correlations and plots illustrating the linear and smoothed associations, respectively, between improvements in determinants (measurement-to-measurement), performance objectives, improvements in SB (week-to-week), and improvements in QoL (measurement-to-measurement). Abbreviations: PBC, perceived behavioural control; PSS, perceived social support; PercSusc, perceived susceptibility; SB CoDA, SB proportion; SSSB, summed squared sitting bouts; TP, task performance; CP, contextual performance; EWB, emotional well-being. ***r > .50; **r > .40; *r > .30

Figure 5 shows the correlations and the univariate distributions of the variables.

#### Correlations Within the Parts of the Logic Model of the Intervention

Among the sub-group, improvement in PBC was positively associated with improvement in attitude (*r* = .46; 95% CI = .15, .68; *p* < .01; *p*_corrected_ = .03) and in intention (*r* = .45; 95% CI = .14, .68; *p* < .01; *p*_corrected_ = .04), which was also found when analysing all participants. Average monitoring delay was negatively associated with average SB registering (*r* =  − .65; 95% CI =  −.78, −.45; *p* = *p*_corrected_ < .001), which was also found for the entire group. Improvement in vitality was positively associated with improvement in emotional well-being (*r* = .59; 95% CI = .33, .77; *p* < .001; *p*_corrected_ = .001), and improvement in perceived stress was positively associated with improvement in emotional well-being (*r* = .58; 95% CI =  −.76, − 0.30; *p* < .001; *p*_corrected_ < .01). All associations within improvements in QoL were also found when analysing all participants.

#### Correlations Between the Parts of the Logic Model of the Intervention

After the Benjamini–Hochberg correction, none of the improvements in one part of the logic model was associated with improvements in another part of the model among the sub-group.

## Discussion

The purpose of this study was to explore moderators of the effectiveness of the UPcomplish intervention, which had previously been found neither to have effects on SB, on psychosocial determinants, nor on QoL [16]. Expectedly, we found that baseline psychosocial determinants and baseline QoL factors were negatively associated with improvements in determinants and QoL. Since baseline determinants and QoL were high among the participants of this study, we conducted a post hoc analysis to investigate whether participants starting lower in determinants and QoL profited from the UPcomplish intervention. Among this sub-group, improvement in PBC was associated with improvement in prolonged sitting, which itself was related to improvement in task performance (see Fig. 5). Yet, these were not significant anymore after the Benjamini–Hochberg correction.

We hypothesized that baseline characteristics of the sample such as psychosocial determinants, working tasks, or QoL would predict intra-individual improvements when receiving UPcomplish. In line with previous research, we had a selective sample majorly including female participants [37, 38], and participants with high intentions to reduce their SB [39]. Additionally, the sample of the current study had higher baseline attitude, PBC (i.e. self-efficacy), and perceived social support as opposed to previous SB intervention studies [40, 41]. Participants showed very high values in perceived susceptibility to too much sitting, which has also been found previously [38, 41]. Beyond showing the selectivity of the sample, these high baseline values might have caused ceiling effects such as baseline intention being associated with lower improvement of intention. None of the determinants was associated with improvements in SB or in the performance objectives (e.g. monitoring behaviour). Since this was found neither with the original sample nor in the post hoc analysis using participants lower in baseline determinants, the psychological determinants per se might not be enough to predict improvements in SB. This is in line with previous research that did not find psychosocial determinants to be mediators for improvements in SB [40].

UPcomplish was mainly aimed at influencing attitudinal, normative, and control beliefs. This might have resulted in too much focus on creating an intention rather than translating the intention into actual behaviour. Others already suggested that the challenge of reducing SB is rather the volitional process, which is one way to bridge the gap between the intention and the actual behaviour [42]. Volition can be promoted (1) by action planning, which includes goal setting and the anticipation of barriers of behavioural change, and (2) by PBC, which elsewhere was already found to be a moderator in reducing workplace SB [40, 42]. Similarly, in the post hoc analyses, we found that among a sub-group of participants scoring lower in baseline determinants, improvement in PBC was the only factor that was marginally related to improvement in SB. Although the UPcomplish intervention did include goal setting, the anticipation of barriers, and several tips aiming at an increase of PBC, the participants did not report an increase in PBC nor did they improve their SB [16]. However, at baseline, the participants had a median score of 4.0 out of 5.0 on PBC, which might have been one of the core reasons for the lack of effectiveness. Therefore, the UPcomplish intervention might only be effective for office workers scoring low in PBC at baseline. Additionally, SB might be less of a reasoned action and more a behaviour that is determined by automaticity and environmental conditions. To break the automaticity of SB, it might be combined with environmental changes (e.g. implementing standing desks, cue altering), and methods to change habits (e.g. reminders). Additionally, support from employers or other leaders might also help bridge the intention-behaviour gap. These could serve as role models, support standing and walking meetings, or implement policies that allow for breaks in sitting time.

Except for perceived vitality, the sample of this study indicated having good QoL, which could be due to a selectivity bias. However, there is no evidence that health affects participation in workplace health interventions [37, 39]. Hence, concerning QoL, the sample of this study might be representative of the working population in Germany. Additionally, although some aspects of QoL at baseline were associated with improvements in QoL during the intervention, they were likely be caused by ceiling effects because they did not relate to the performance objectives, or to improvements in psychosocial determinants and in SB [43]. Only in the post hoc analysis among a sub-group, we found a tendency that improvement in perceived physical pain was associated with fewer reductions of SB. However, this was not significant after the Benjamini–Hochberg correction. In another study, lower back pain at the beginning of the intervention predicted less improvement in SB, which was assumed to be caused by a limited capacity of standing due to the perceived pain [44].

Several steps of the implementation of the UPcomplish intervention might increase its effectiveness. Firstly, increasing the reach by also including employees being less motivated and self-efficacious at baseline could improve its effectiveness considering the focus of UPcomplish being on psychosocial determinants. To overcome challenges in the adoption of workplace health programs [45], a systematically developed implementation plan using Implementation Mapping might help to increase the reach of UPcomplish [46]. For example, it would be important to increase awareness of the program, self-efficacy towards participation (e.g. to overcome time constraints and tiredness), and attitudes regarding the program among all potential participants already before they potentially adopt the program [45]. Secondly, although the acceptability, understandability, and the message processing of the UPcomplish intervention were positive, more components need to be included to address other ecological levels [15, 37]. Multi-component interventions have the potential for higher adoption rates due to an increased likelihood to match with the needs of participants [37]. Additionally, a workplace SB intervention including a psychosocial intervention, but also managers serving as role models, financial incentives to increase sustained participation, and environmental (e.g. standing desks) and cultural (e.g. walking around is seen as healthy and not as time-wasting) restructuring is likelier to be effective on the long run because it tackles both automatic and controlled motivational processes [41, 47, 48]. Lastly, although the UPcomplish intervention was systematically developed using the IM framework [13], the intervention content might not have tackled all important psychosocial determinants [49]. This should be investigated within the scope of a process evaluation implementing the intervention among participants with low baseline determinants.

### Strengths and Limitations

This study has several strengths. First, we had longitudinal data of diverse company industries to our disposal that were collected during 75% of an entire year, and we additionally accounted for seasonal variations by centring the variables around calendar week means. A cheap and unobtrusive measurement tool with long battery life, the VitaBit device, facilitated the continuous collection of SB data. This increases the external validity of the results. Second, we were the first to our knowledge that incorporated information on the entire logic model of a SB intervention, which provides interesting insights into the underlying mechanisms of reducing workplace SB. Third, we focused on the health effects for the target group, which was the reason to analyse SB during the entire day and not merely SB during workdays. Fourth, not the absolute time spent sitting but rather prolonged, uninterrupted SB is associated with detrimental health outcomes. Hence, we applied both a compositional data approach to account for inter-dependencies of physical behaviours and a new value (i.e. SSSB) to represent prolonged SB. Last, UPcomplish was highly accepted among participants: the participants did not only indicate that they perceived the intervention positively also did they drop out late and mostly if they had technical problems rather than if they lost their motivation [50].

One of the limitations is that, since the psychosocial determinants and QoL were measured using self-reports, participants might have provided socially desirable answers [51]. However, concerning QoL, using self-reports enabled the assessment of a large number of participants with lower timely and financial resources. Another limitation concerns the employees that did not adopt the intervention. Voluntary participation might have resulted in a selection bias, and our sample included mainly females, and participants scoring high in psychosocial determinants at baseline. However, we conducted a post hoc analysis to investigate potential effects among a sub-group scoring lower in the psychosocial determinants. Lastly, we could not cluster the data by company using multilevel models nor could we centre the variables in the post hoc analysis around calendar week means because this would have resulted in fewer data, and therefore, less statistical power and a problem of singularity.

### Conclusions

Especially high baseline values in, for example, intention were negatively related to intra-individual improvement in the intention to sit less. However, this study showed that, except for PBC, the psychosocial determinants (attitude, perceived social norms, perceived susceptibility, intention) do not seem to be important when reducing workplace sitting, and it might be more determined by the organizational environment and automatic behaviours. When promoting health at the workplace, it is a challenge to reach a representative sample of employees including the ones being less interested in improving their health. Yet, this study showed, that probably especially these employees could profit most from a motivational intervention. It needs to be investigated whether UPcomplish could be effective in combination with changes in the physical and cultural environment of companies.

## Data Availability

The cleaned raw data and additional material is fully disclosed in the supplementary materials: https://osf.io/qzp9m/?view_only=30ada8d6fc0e4ac19a1610b8901f9f96.

## Abbreviations

SB: Sedentary behaviour
QoL: Quality of life
PBC: Perceived behavioural control
BMI: Body mass index,
MVPA: Moderate-to-vigorous physical activity
IM: Intervention Mapping
